# On the Morphology of the Digestive System of Two *Monomorium* Ant Species

**DOI:** 10.1673/031.013.7001

**Published:** 2013-07-15

**Authors:** Daniel Russ Solis, Mônica Lanzoni Rossi, Eduardo Gonçalves Paterson Fox, Neusa de Lima Nogueira, Francisco André Ossamu Tanaka, Odair Correa Bueno

**Affiliations:** 1 Centro de Estudos de Insetos Sociais, Instituto de Biociências, São Paulo State University, Rio Claro, São Paulo, Brazil; 2 Laboratório de Histopatologia e Biologia Estrutural de Plantas, Centro de Energia Nuclear na Agricultura, University of São Paulo, Piracicaba, São Paulo, Brazil; 3 Instituto de Biofísica Carlos Chagas Filho, Federal University of Rio de Janeiro, Rio de Janeiro, RJ, Brazil; 4 Departamento de Fitopatologia e Nematologia, University of São Paulo, Piracicaba, São Paulo, Brazil

**Keywords:** Formicidae, *Monomorium floricola*, *Monomorium pharaons*, Myrmicinae, Solenopsidini, tramp species

## Abstract

The digestive system of adults and mature larvae of two ant species of *Monomorium* Mayr (Hymoneptera: Formicidae) were described with the aid of light and scanning electron microscopy, as there is a lack of studies in this area. These two ant species are recurrently found in urban habitats and are known as ‘tramp species,’ as they cause problems in households, businesses, and hospitals. The most interesting finds of the present study include the existence of spinules in the crop of adults, and the number of Malpighian tubules and rectal pads was constant among different castes, ages, and species.

## Resumo

O sistema digestório de adultos e larvas maduras foi descrito em duas espécies do gênero *Monomorium* Mayr (Hymoneptera: Formicidae), com auxilio de microscopia óptica e eletrônica de varredura, para suprir uma carência no conhecimento sobre esse assunto, para esse grupo. Estas duas espécies são comumente encontradas em ambiente urbano, conhecidas pelo nome genérico de ‘tramp species’, que podem ocasionar problemas em residências, indústrias e hospitais. Como resultados interessantes foram verificados a existência espinhos no papo dos adultos, e um número não variável de túbulos de Malpighi e papilas retais entre as castas, idades e espécies.

## Introduction

The digestive system of adult insects is divided into three regions (and subdivisions): the foregut (pharynx, esophagus, crop, and proventriculus), midgut, and hindgut (pylorus, ileum, and rectum) (Chapman 1998). Although the digestive systems of different insect groups are generally alike, there are morphological specializations for different feeding habits (Caetano 1984).

Most ants are omnivorous species, and adults feed from liquids while larvae feed from either liquid or solid food, depending on the species. In many species, food and gland secretions are shared among the colony members, a practice termed ‘trophollaxis.’ Because of this practice, the digestive system of each individual ant plays an important role in the social organization of its colony (Fowler et al. 1991).

The morphological variation of some parts of the digestive systems of ants can be useful to describe and sort between taxonomic groups. For instance, the shape of the proventriculus may vary, and in some groups the esophagus of females (workers and queens) may present unique dilations; also, the ileum may be dilated and contain cuticular spinules, and the number of rectal pads can also vary (Caetano 1990; Caetano et al. 1990).

The ant genus *Monomorium* Mayr (Hymoneptera: Formicidae) comprises 399 described species (Bolton et al. 2006), including species of cosmopolitan distribution that are considered pests (also termed ‘tramp species’), such as *Monomorium floricola* Jerdon and *Monomorium pharaonis* L. (Bolton 1987). Despite the importance of this ant genus, there are few studies on the morphology of the digestivesystem of this taxon. Petersen-Braun and Buschinger (1975) verified the esophagus of adult queens of *M. pharaonis* had dilations that served functions similar to the crop. In a revisional study of *M. pharaonis*, Berndt and Eichler (1987) presented an illustration of the internal morphology of adult ants, in which four Malpighian tubules and two rectal pads were drawn. Concerning ant larvae, two studies (Athias-Henriot 1947; Valentini 1951) described their internal morphology, including *Monomorium ajjer* Bernard, *Monomorium salomonis* L., and *Monomorium subopacum* Smith. These authors reported the existence of four Malpighian tubules in larvae of these *Monomorium* species.

Towards a better understanding of *Monomorium*, the present study aimed at describing the digestive system of *M. floricola* and *M. pharaonis*, using light and scanning electron microscopy to do so. The finds might have important implications in physiological and comparative morphology studies.

## Materials and Methods

Ten specimens of *M. floricola* and *M. pharaonis* belonging to different life stages and castes (last instar larvae and adults of queens, males and workers) were observed by light and electron microscopy, totalling 80 analyzed specimens of each species. The digestive system of the ants was described both qualitatively and quantitatively. Mature larvae were sorted following the methods explained by Alvares et al. (1993) and Solis et al. (2010a). According to these authors, *M. floricola* and *M. pharaonis* have three larval instars.

Morphological descriptions of the digestive system of larvae and adults are given separately, following the terminology of Petralia and Vinson (1980), Caetano (1988), Landim (2008). Malpighian tubules were included in the descriptions, although they actually belong to the excretory system, and were measured and counted. The following procedures describe sample preparation.

### Light Microscopy

Specimens were dissected under a stereomicroscope on a glass slide with saline solution. Digestive systems were mounted and examined under a light microscope Zeiss MC80 DX (maximum magnification 1000x) (www.zeiss.com) equipped with a micrometric eyepiece, and photographed with a Sony Cyber-Shot DSC-W210 digital camera (www.sony.com). Whenever possible, measurement figures are presented as means plus standard deviation, followed by the minimum and maximum interval and the number of observations (n). To assess differences between the two species, Student's *t*-test was employed (*p* < 0.05). Moreover, in order to check for statistical intraspecific differences in the size of spinules inside the crop of the two species, regarding sex and castes, analysis of variance (ANOVA) with Tukey's test (*p* < 0.05) was performed.

Some samples were placed in HistoResin (Leica Biosystems, www.leicabiosystems.com) for histological analysis. As the morphology of the digestive system of both species was extensively similar, only samples of *M. floricola* were included and mounted. These were fixed (3% glutaraldehyde, 0.005 M sodium cacodylate, 1.8% sucrose) for 48 hr and dehydrated in a graded series of ethanol concentrations (30%, 50%, 70%, 90%, and 100%; 10 min-dips, last concentration used thrice). After dehydration, samples were left for 24 hr in 1:1 ethanol Leica resin, and then embedded for 24 hr in pure Leica resin. Then, the resin containing samples was added to a hardener and left to dry in molds. Resulting blocks were sliced with a rotational microtome Leica RM 2155, producing 0.005-mm-thick sections. Sections were stained with 0.05% toluidine blue. Sections were attached to glass slides with Entelan (Merck, www.merck.com), and were examined and photographed under a Zeiss Axioskop 40 HBO 50 light microscope (maximum magnification 1000x).

### Scanning electron microscopy

Samples were dissected and dehydrated as explained, and critical-point dried with a Balzers CPD/030(www.oerlikon.com/balzers). Dry samples were attached to aluminium stubs with double-faced adhesive tape. Dry larvae were gently cut open while on the stubs to expose their digestive tubes. All samples were then coated with gold with a Balzers SCD/050 ion sputterer. Observations and pictures were taken with the scanning electron microscope LEO 435 VP at 20.0 kV (NAP/MEPA, ESALQ, University of São Paulo).

## Results

Measurements of the digestive systems of *M. floricola and M. pharaonis* are shown in Table 1. A joint morphological description is given below, yet separated for larvae and adults.

### Last instar larvae

**Figure 1.**
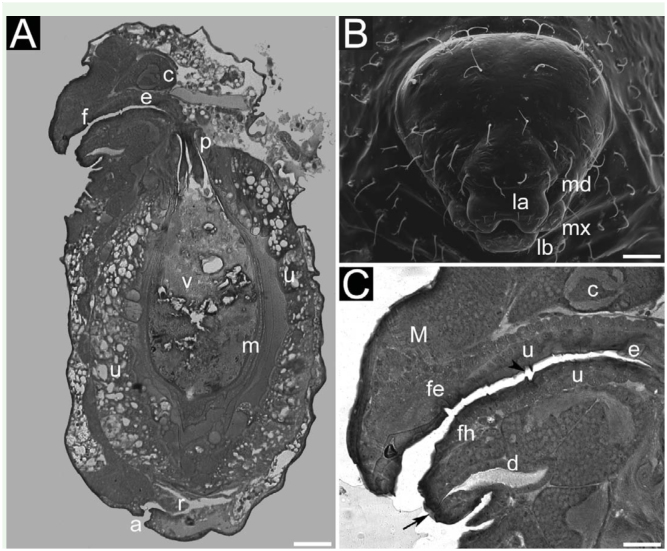
Digestive system of last-instar larvae of *Monomorium floricola:* (A) longitudinal section of whole body; (B) frontal view of head capsule; (C) closer view of anterior region. Figures 1A and 1C are light micrographies; figure 1B is scanning electron micrographies. Abbreviations: anus (a), brain (c), common duct (d), epipharynx (fe), epithelium (u), hypopharynx (fh), labium (lb), labrum (la), mandibles (md), maxilla (mx), muscles (M), oesophagus (e), pharynx (f), peritrophic envelope (m), proventriculus (p), rectum (r), spinules (black arrowhead), valve of labial gland system (black arrow), ventriculus (v). Size of scale bars: (A) 0.081 mm, (B) 0.037 mm, (C) 0.034 mm. High quality figures are available online.

For both species, the structures with greater and lesser lengths were respectively the Malpighian tubules and rectal pads. Excepting the rectum, all structures were longer in specimens of *M. pharaonis*. Figure 1A displays a general panorama of the digestive system of a mature larva of *M. floricola*. As mentioned, the digestive systems of both species were quite similar.

**Figure 2.**
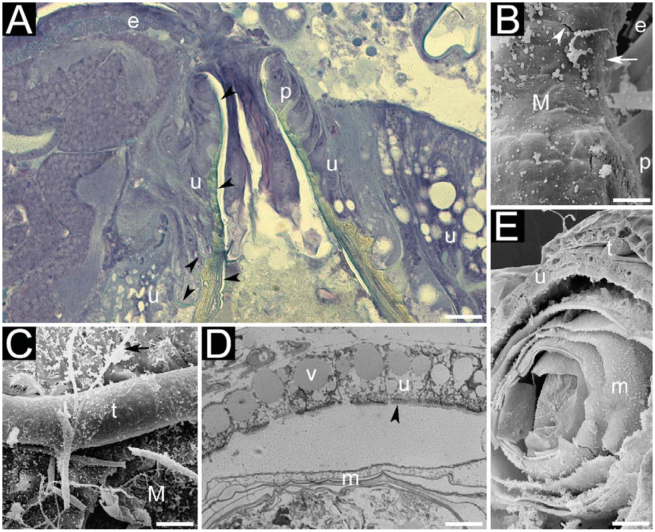
Digestive system of last-instar larvae of *Monomorium floricola:* (A) transitional region between foregut and midgut, showing the formation of the peritrophic membrane (black arrowheads); (B) external surface of foregut; (C) external surface of ventriculus; (D) section of internal wall of ventriculus; (E) transversal section of body, showing ventriculus. Figures 2A and 2D are light micrographies; figures 2B, 2C, and 2E are scanning electron micrographies. Abbreviations: circular musculature (white arrowheads), epithelium (u), longitudinal muscles (white arrows), Malpighian tubules (t), microvillosities (black arrowhead), muscles (M), oesophagus (e), peritrophic envelope (m), proventriculus (p), tracheoles (black arrows), vesicles (v). Size of scale bars: (A) 0.031 mm, (B) 0.010 mm, (C) 0.014 mm, (D) 0.044 mm, (E) 0.050 mm. High quality figures are available online.

Foregut. Comprising oral cavity, pharynx, esophagus, and proventriculus. Oral cavity formed by epipharynx, hypopharynx, mandibles, and maxilla (sitting on the sides) (Figures 1B, 1C). Pharynx presenting spinules (Figure 1C). Esophagus an extension of the pharynx, being a long and slightly inclined tube with no dilation (Figure 1A). Proventriculus was slightly dilated. Foregut internally lined with an epithelium lined with apical cuticle, sitting on a double layer of visceral muscles—longitudinal muscles standing on circular muscles (Figure 2B). The extrinsic muscles (Figure 1C) sustain the pharynx. These structures were transparent when seen with the light microscope.

Midgut. Ventriculus white and rounded, with the peritrophic envelope inside; the latter being yellowish and elliptical, with many laminae (Figure 2E), the last lamina containing ingested food. Epithelium containing welldefined prismatic cells (main cell type, also termed enterocytes) (Figure 2D) and filiform cells (Figure 2A). Prismatic cells about 0.050 mm high, lined with microvillosities instead of cuticle (Figure 2D). Laminae are formed by the epithelium of the proventriculus and by the transitional cells from the junction of proventriculus to the ventriculus (Figure 2A). Two types of laminae could be observed (data not shown): thin and thick. Moreover, similar to the observed with the foregut, ventriculus also comprised two layers of visceral muscles.

Hindgut. Formed by a basal region, small intestine, rectum, and the anus. All larvae had four Malpighian tubules connected to the small intestine. Moreover, Malpighian tubules were attached to the ventriculus by a mesh of tracheoles (Figure 2C). The rectum was an ample and transparent sac formed by cuboidaland squamous cells, and presented three ovoid rectal pads.

### Adults

**Figure 3.**
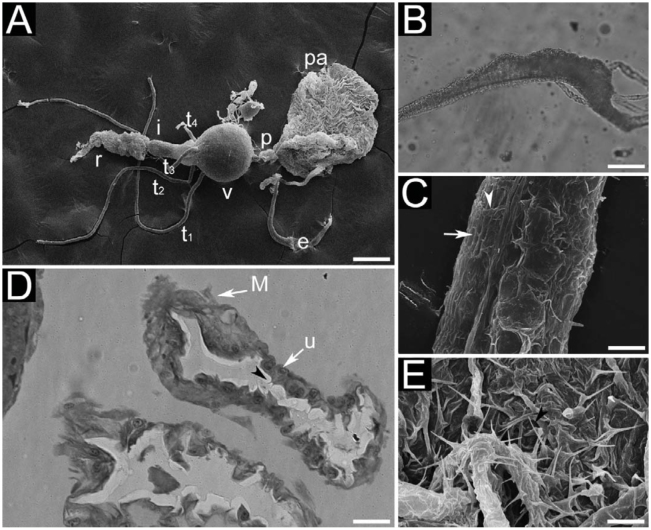
Digestive system of adults of *Monomorium floricola:* (A) digestive of queens; (B) dilation of queen oesophagus; (C) detail on queen oesophagus, showing circular muscles (white arrowhead) and longitudinal muscles (white arrow); (D) longitudinal section of male crop; (E) internal surface of a worker crop. Figures 3B and 3D are light micrographies; figures 3A, 3C, and 3E are scanning electron micrographies. Abbreviations: crop (pa), epithelium (u), ileum (i), Malpighian tubules (tn), muscles (M), oesophagus (e), proventriculus (p), rectum (r), spinules (black arrowhead), ventriculus (v). Size of scale bars: (A) 0.200 mm, (B) 0.053 mm, (C) 0.011 mm, (D) 0.023 mm, (E) 0.010 mm. High quality figures are available online.

Like in larvae, the longest structures were the Malpighian tubules and rectal pads. In queens and workers, the esophagus, proventriculus, ventriculus, rectal pads, and Malpighian tubules were longer in *M. pharaonis*. Also, the rectum of *M. pharaonis* workers was longer than in *M. floricola*. The remaining structures, crop and ileum and rectum of queens, were similar in size for both species. Males of *M. pharaonis* had longer structures than males of *M. floricola*, excepting the ileum, which was of similar size. In general, the digestive systems of males and females were similar, excepting the esophagus of queens (see below). Figure 3A presents a general panorama of the digestive system of *M. floricola* queens.

**Figure 4.**
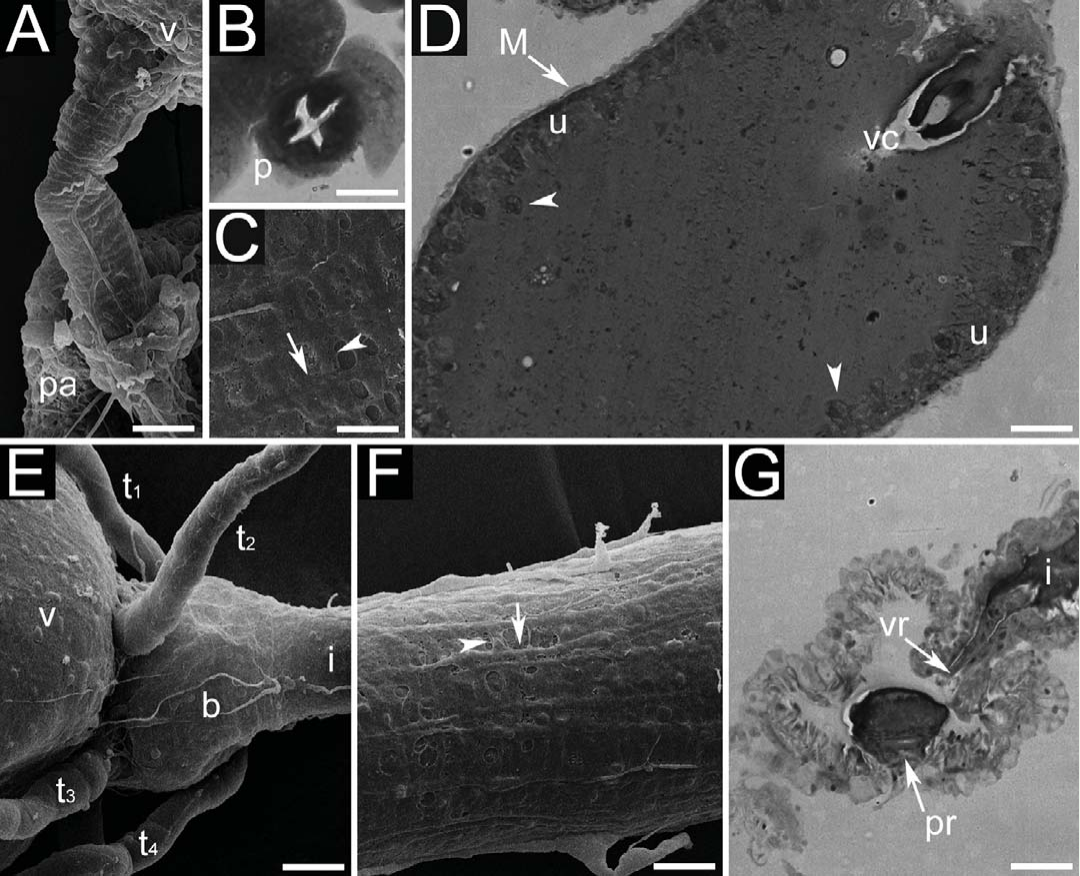
Digestive system of adults of *Monomorium floricola:* (A) queen proventriculus; (B) transversal section of proventriculus of queen; (C) closer view of ventriculus surface of queen; (D) longitudinal section of ventriculus of queen; (E) male pylorus; (F) queen ileum; (G) longitudinal section of queen rectum. Figures 4B, 4D and 4G are light micrographies; figures 4A, 4C, 4E and 4F are scanning electron micrographies. Abbreviations: cardiac valve (vc), circular muscles (white arrowhead), crop (pa), epithelium (u), formation and release of bubbles (black arrowheads), ileum (i), longitudinal muscles (white arrow), Malpighian tubules (tn), muscles (M), proventriculus (p), pylorus (b), rectal pads (pr), rectal valve (vr), ventriculus (v). Size of scale bars: (A) 0.040 mm, (B) 0.012 mm, (C) 0.020 mm, (D) 0.050 mm, (E) 0.020 mm, (F) 0.020 mm, (G) 0.038 mm. High quality figures are available online.

Foregut. Formed by oral cavity, pharynx, esophagus, crop, and proventriculus. In both species, the esophagus was a long tube initiating in the head and extending towards the mesosoma and waist. In queens, the esophagus was dilated inside the mesosoma (Figure 3B). The crop, when completely full, was a hollow round sac, becoming wrinkled when empty (Figure 3A). Inside the crop, the epithelium was formed by short cells lined with a cuticle (Figure 3D) presenting spinules upon connection with esophagus and proventriculus (Figure 3E). Spinule size in *M. floricola* varied with sex and caste. In *M. pharaonis*, spinules were longer in workers than in queens and males, the latter two presenting spinules of similar size. The proventriculus was a short tube, without including the bulb (Figures 4A, 4B). Visceral muscles of foregut were composed of an internal layer of longitudinal fibers and an outer layer of circular fibers (Figure 3C). These three structures were transparent when observed under the light microscope.

Midgut. Ventriculus rounded when full, and appearing light or dark brown under the light microscope, depending on the specimen. The prismatic cells of the epithelium could be easily identified by apocrine secretion of substances (looking like protruding bubbles; Figure 4D) and by their characteristic lining with numerous microvillosities (data not shown). Outside the ventriculum, intermingled external longitudinal and internal circular muscle fibers formed a net (Figure 4C). The peritrophic envelope could not be identified.

Hindgut. Formed by pylorus, ileum, and rectum. The pylorus is a dilation on the digestive system between the ventriculum and ileum, in which the Malpighian tubules converge (Figure 4E). The ileum is a long tube connecting the pylorus to the rectum. Outside the ileum, there were longitudinal muscle fibers sitting on internal circular muscle fibers, thus forming a net (Figure 4F). Four Malpighian tubules were observed disposed in a single row (Figure 4E). In *M. pharaonis*, 10% of the observed specimens (both males and workers) had five Malpighian tubules, and in all cases two of the tubules shared the same connection into the pylorus. The rectum was an ample sac with a thin wall (Figure 4G). Three oval rectal pads were found, near the proximal region of the rectum.

## Discussion

Rows of spinules in the pharynx (hypopharynx) of different ant larvae were recently reported by other authors (e.g., Jesus et al. 2010; Solis et al. 2010b; Nondillo et al. 2011), but were curiously not reported in *M. pharaonis* by Petralia and Vinson (1980). Wheeler and Wheeler (1976) reported that 70% of their examined larvae of different ant species had spinules in the hypopharynx, yet in varying amounts and occurring either in rows or isolated. They guessed the spinules were probably related with food trituration.

Chapman (1998) mentioned that the crop of insects presented folds in the walls when empty, thus being apparently extensible to accommodate incoming food and serving as a storage unit. According to the same author, many insects have cuticle projections in the crop that look like spinules and denticles, which might play a role in driving the food towards the midgut. In hymenopterans like bees and ants, the crop serves as a food storage unit for the whole colony, as it enables food to be passed on to other colony members (Caetano 1984, 1988, 1990; Landim 2008); however, cuticular projections in the crop were never reported. Yet, cuticular projections were found in other parts of the digestive system of hymenopterans. There are spines in the esophagus of *Solenopsis richteri* Forel queens, which can be found down to the connection with the crop (Walker and Clower 1961). In the proventriculus of bees and ants, there are spines that apparently sift the ingested food coming towards the ventriculus (Caetano 1984; Landim 2008; Solis et al. 2009). The ileum of some ant species may contain spinules, which are believed to also aid in pushing the ingested food (Caetano 1990). We thus think the spinules observed in *Monomorium* must also serve the purposes of sifting and moving food down the digestive system from the crop.

The crop was relatively larger in workers than in queens and males (Caetano 1984; Landim 2008), probably reflecting the main roles of worker ants in colony nutrition, namely food collection, transportation, and distribution (Hölldobler and Wilson 1990). However, the crop was never larger in workers when the relative size contribution to the whole digestive system was considered (*M. floricola*: workers 19%; queens 20%; males 12%; *M. pharaonis:* workers 15%; queens 16%; males 14%). In both species, crops of females were of equivalent relative size, while the crops of *M. pharaonis* males were about the same size as the females’. During parallel food preference tests (unpublished results), queens were often observed participating in the foraging trails, sometimes even collecting food. The participation of queens in the foraging trails of workers did not look like some laboratorydriven behavior deviation, since Forel (1893) also reported the presence of queens in the foraging trails of *M. floricola*. Moreover, in both species of *Monomorium*, queens had an esophagus dilation forming a ‘thoracic crop,’ which was already reported in *Monomorium*. It is possible that this dilation is complementary to the normal function of the crop, as it is compressed inside the gaster by the developing ovary (Caetano 1990). Thus, queens and males of *Monomorium* would not only be restricted to reproductive functions, but also play active roles in food collection and storage. As an example of this secondary task, Hölldobler and Wilson (1990) reported males of *Camponotus* passing food on to the other members of the colony.

The peritrophic membrane could be easily observed in larvae and presented a double origin: epithelium of the proventriculus and the transition cells between the ventriculus and proventriculus. In addition, Landim (2008) mentioned a third formation site for peritrophic membranes: at the apex of the epithelium cells of the midgut. Although this was not observed in the present investigation, it may have been overlooked. No peritrophic membrane was observed in adults. However, it may have been overlooked, as it has been difficult to spot by light microscopy in observations of other ants (Caetano 1990).

Landim (2008) described the existence of four cell types in the ventriculus of bee larvae: the main prismatic cells (with a primary digestive function), the regenerative cells, the filiform cells (involved in the synthesis of some parts of the peritrophic membrane and located on the transition between foregut and midgut), and the endocrine cells. In the present investigation, only the main prismatic cells and filiform cells were found, yet the existence of the other cell types should not be discarded. Moreover, Landim (2008) also reported the existence of four types of cells in the ventriculus of adult bees: prismatic cells surrounding the cardiac valve and the main, and basal cells being either regenerative or endocrinous. However, Caetano (1990) mentioned the existence of only two cell types in the ventriculus of adult ants: digestive and generative. In the present investigation, only prismatic cells were found, which according to Landim (2008) are digestive cells that absorb the digestion products and make up most of the local epithelium. The existence of other, less frequent cell types ought not to be dismissed, because even though they were not observed, they most likely would only show in further observations and with the aid of other techniques (e.g., transmission electron microscopy).

Caetano (1984, 1988) said that the number of Malpighian tubules in adult ants would vary both interespecifically and intraspecifically (i.e., between different castes). Caetano (1988) also reported the existence of a direct correlation between the size of the adult ant and the internal number of Malpighian tubules, with smaller ants having fewer tubules. According to Landim (2008), the number of tubules in bee larvae and adults was different in the same species. By comparing these statements with the results observed in the present study, we concluded they are not true in the present case; the number of Malpighian tubules remained fixed among ants of different species, size, caste, and ages. It is a curious fact that Petralia and Vinson (1980) stated that ant and bee larvae would always have four Malpighian tubules. However, Landim (2008) stated that four tubules would be the most frequent number in bee larvae, but this figure could vary between four and twelve. In light of the more recent information about bees, it seems plausible that the number of tubules among ant larvae is also variable; however, the number inside the observed specimens remained the same.

Landim (2008) mentioned that some bees of the genus *Melipona* presented a positive correlation between size of the adult and the length of the Malpighian tubules, with larger specimens presenting longer tubules. This result was the same as our study, as the Malpighian tubules of *M. pharaonis* were longer than the tubules of smaller *M. floricola*. Caetano (1984) mentioned that the number of Malpighian tubules would always be a multiple of two. This fact agrees with the observations of *M. floricola*, but there were exceptions in *M. pharaonis*, as 10% of the observed specimens had five tubules. Could this exception be the result of malformation? Landim (2008) found adult workers in the bee *Nannotrigona testaceicornis* Lepeletier had 25 Malpighian tubules, in spite of the fact that other species had paired numbers of tubules.

According to Caetano (1988), the number of rectal pads of adult ants should remain constant among ant subfamilies, e.g., with Myrmicinae always presenting three pads, as also observed in the present study for the two species of *Monomorium*. With ant larvae, Petralia and Vinson (1980) mentioned the existence of three rectal pads, a result that was confirmed in the present study. However, as mentioned by Solis et al. (2009) with *P. longicornis*, there are exceptions to the number of rectal pads, and thus we wondered if the number in *Monomorium* would be variable.

The present study described the digestive system of *Monomorium* based on numerous specimens of two important tramp species in an attempt to cover both interspecific and intraspecific variation. Some traits unique to this group of ants were illustrated, like the spinules inside the crop of adult ants and the fixed number of rectal pads and Malpighian tubules in adult and immature stages. The results were compared with previous findings of other authors, either confirming their assumptions or proposing new hypotheses. Further studies with other related species and electron transmission microscopy will certainly add to the herein presented overview.

**Table 1.**
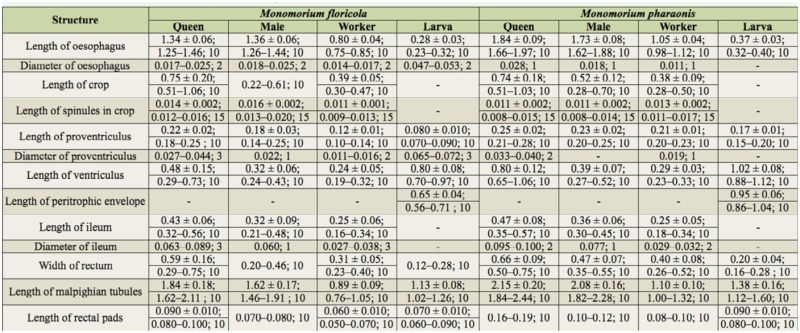
Measurements (in mm) of different structures of the digestive systems of two *Monomorium* ant species. Results presented as follows: means ± standard deviation (whenever possible); minimum and maximum interval; number of observations
